# Maternal prepregnancy overweight/obesity increase the risk of low Apgar scores in twins: a population-based cohort study in China

**DOI:** 10.3389/fped.2024.1412975

**Published:** 2025-01-17

**Authors:** Zhoushan Feng, Xiaohong Wu, Xiaomei Tong, Zheng He, Chunxia Yang, Wei Shen, Yueqin Ding, Jin Liu, Qiong Meng, Aiqin Zhang, Hong Jiang, Wenkang Yan, Jianwu Qiu, Xian Wei, Yayu Zhang, Xiaobo Lin, Lijun Liu, Ya Jin, Youfen Wei, Xiufang Yang, Yitong Wang, Yangfan Cai, Xinzhu Lin, Qiliang Cui

**Affiliations:** ^1^ Department of Neonatology, Guangzhou Key Laboratory of Neonatal Intestinal Diseases, The Third Affiliated Hospital of Guangzhou Medical University, Guangzhou, China; ^2^ Department of Obstetrics and Gynecology, Guangdong Provincial Key Laboratory of Major Obstetric Diseases, The Third Affiliated Hospital of Guangzhou Medical University, Guangzhou, China; ^3^ Department of Pediatrics, Peking University Third Hospital, Beijing, China; ^4^ Department of Neonatology, Sichuan Jinxin Xinan Women & Children’s Hospital, Chengdu, China; ^5^ Department of Neonatology, The Affiliated Hospital of Guizhou Medical University, Guiyang, Guizhou, China; ^6^ Department of Neonatology, Xiamen Maternal and Child Health Care Hospital, Xiamen, China; ^7^ Department of Neonatology, Affiliated Dongguan Shilong People’s Hospital of Southern Medical University, Dongguan, Guangdong, China; ^8^ Department of Neonatology, First Affiliated Hospital of Shaoyang University, Shaoyang, China; ^9^ Department of Neonatology, Guangdong Second Provincial General Hospital, Guangzhou, Guangdong, China; ^10^ Department of Neonatology, Cangzhou People’s Hospital, Cangzhou, Hebei, China; ^11^ Department of Neonatology, Affiliated Hospital of Yanan University, Yan an, China; ^12^ Department of Neonatology, Huizhou Central People’s Hospital, Huizhou, China; ^13^ Department of Neonatology, The Affiliated Yue Bei People’s Hospital of Shantou University Medical College, Shaoguan, China; ^14^ Department of Neonatology, Xiaogan Hospital Affiliated to Wuhan University of Science and Technology, Xiaogan, China; ^15^ Department of Neonatology, The Affiliated Hospital of Inner Mongolia Medical University, Hohhot, China; ^16^ Department of Neonatology, The Second Affiliated Hospital of Shantou University Medical College, Shantou, China; ^17^ Department of Neonatology, The Third Staff Hospital of Baogang Group Baotou, Baotou, China; ^18^ Department of Neonatology and Pediatrics, Jinan University First Affiliated Hospital, Guangzhou, China; ^19^ Department of Neonatology, Lanzhou University Second Hospital, Lanzhou, China; ^20^ Department of Neonatology, Zhongshan City People’s Hospital, Zhongshan, China; ^21^ Department of Neonatology, Binhaiwan Central Hospital of Dongguan, Dongguan, China; ^22^ Department of Neonatology, The First Affiliated Hospital of Shantou University Medical College, Shantou, China

**Keywords:** pregnancy, twins, Apgar score, overweight, neonatology

## Abstract

**Objective:**

While prepregnancy overweight or obesity is known to negatively impact maternal health, its effect on twin infants is not well understood. Therefore, we conducted a nationwide, multicenter retrospective study to investigate the association between maternal prepregnancy weight and health outcomes in twins.

**Study design:**

This study collected data from 22 healthcare units across 12 regions in China between January 2018 and December 2020. To control for confounding factors, multiple logistic regression, propensity score matching (PSM), inverse probability of treatment weighting (IPTW), and overlapping weighting models (OW) were applied to explore the effects of prepregnancy BMI on Apgar scores and other outcomes.

**Results:**

After screening, a total of 4,724 women with twin pregnancies and 9,448 newborns were included in the study. Compared to normal prepregnancy weight, prepregnancy overweight/obesity significantly increased the risk of gestational hypertension and gestational diabetes in mothers [adjusted OR (95% CI): 1.85 (1.55–2.21) and 1.49 (1.27–1.74), respectively]. It also increased the incidence of twins with a 1-min Apgar score ≤7, whether they were larger or smaller [1.60 (1.20–2.13) and 1.45 (1.09–1.92), respectively]. Sensitivity analyses using PSM [1.60 (1.20–2.13) and 1.55 (1.07–2.25)], IPTW [1.67 (1.31–2.12) and 1.48 (1.17–1.87)], and OW [1.65 (1.08–2.57) and 1.47 (0.97–2.25)] confirmed the stability of these results. However, it did not affect the likelihood of a 5-min Apgar score ≤7 [adjusted OR (95% CI): 0.82 (0.24–2.17) and 1.40 (0.70–2.73)]. In contrast, prepregnancy underweight was associated with a reduced incidence of twins with a 1-min Apgar score ≤7 [adjusted OR (95% CI): 0.56 (0.32–0.92) and 0.58 (0.34–0.94)], but had no effect on the 5-min Apgar score ≤7 [adjusted OR (95% CI): 0.82 (0.24–2.17) and 0.22 (0.01–1.08)]. Prepregnancy BMI did not significantly affect twin birth weight discordance, NICU admission, preterm birth, or low birth weight.

**Conclusion:**

Maternal overweight/obesity before pregnancy increases the risk of hypertensive disorders and gestational diabetes in twin pregnancies and significantly raises the likelihood of twins having a low 1-min Apgar score. However, no significant impact on 5-min Apgar scores was observed. These findings highlight the importance of managing weight before pregnancy and ensuring readiness for neonatal resuscitation during delivery.

## 1 Introduction

Maternal overweight and obesity have become pressing global public health concerns, with rates showing a troubling increase in recent years (1). In China, about 17% of adults are classified as obese (2). Maternal overweight and obesity are associated with a greater risk of adverse pregnancy outcomes, such as gestational diabetes (GDM), preeclampsia, preterm birth, and macrosomia (3, 4). These conditions also correlate with elevated risks of neonatal morbidity and mortality (5).

The Apgar score is a widely used assessment tool that evaluates newborns' physical condition immediately after birth based on five key indicators: heart rate, respiratory effort, muscle tone, reflex response, and skin color. A low Apgar score at 1 or 5 min is linked to an increased risk of neonatal asphyxia, long-term developmental disabilities, cerebral palsy, and even neonatal mortality (6, 7). Several studies have investigated the relationship between maternal prepregnancy BMI and Apgar scores in singleton pregnancies (5, 8); however, there is limited evidence regarding twin pregnancies. Twin pregnancies inherently carry a higher risk of adverse outcomes than singleton pregnancies (9). Additionally, research on this subject in China is scarce.

This study aimed to investigate the impact of prepregnancy BMI on outcomes in twin pregnancies, focusing on the 1-min Apgar score. We also examined effects on the 5-min Apgar score, as well as the risks of preterm birth, low birth weight (LBW), NICU admission, and birth weight discordance in twins (BWDT). Our goal is to provide insights that can support improved prepregnancy weight management and early risk assessment during delivery.

## 2 Materials and methods

### 2.1 Study population

This study retrospectively analyzed data collected from 22 medical centers across 12 provinces, municipalities, or autonomous regions in China between January 2018 and December 2020. Trained investigators extracted data from electronic medical records for pregnant mothers and their twins. The Third Affiliated Hospital of Guangzhou Medical University served as the coordinating center, overseeing data review, integration, and analysis. Missing prepregnancy BMI data were excluded during data cleaning. To minimize confounding factors, cases with chromosomal abnormalities, twin-to-twin transfusion syndrome, twin anemia-polycythemia sequence, hydrops fetalis, and preconception diabetes were also excluded.

### 2.2 Outcomes and definitions

The primary outcome were 1-min and 5-min Apgar score ≤7, which serve as key indicators of neonatal asphyxia. Secondary outcomes included NICU admission, BWDT ≥20%, preterm birth, and LBW. Maternal prepregnancy BMI was categorized as underweight (<18.5 kg/m^2^), normal weight (18.5–23.99 kg/m^2^), and overweight/obese (24–27.99 kg/m^2^ or ≥28 kg/m^2^) based on the Chinese standards (10). Pregnancy weight gain (PWG) was categorized as adequate or inadequate according to the Institute of Medicine 2009 guidelines (11). Women aged 35 years or older were considered to have advanced maternal age. Mode of conception was categorized as natural or assisted, including artificial insemination and *in vitro* fertilization with embryo transfer. Abnormal thyroid function included both hypothyroidism and hyperthyroidism. Gestational hypertension and diabetes were diagnosed following national guidelines (12, 13), while adverse pregnancy history encompassed stillbirth and miscarriage.

### 2.3 Covariates

When analysing the association between prepregnancy BMI and neonatal outcomes and complications during pregnancy, potential confounding factors were considered, such as maternal age, hypertensive disorders, mode of conception and delivery, parity, adverse pregnancy history, thyroid function, infections, anaemia, chorionicity, GDM, PWG, and ethnicity. Definitions and categorizations of these covariates are detailed in Supplementary Table S1.

### 2.4 Ethical approval

This nationwide multicentre study was approved by the Ethics Committee of the Third Affiliated Hospital of Guangzhou Medical University with the approval number: CERP [2020] No. 097. The study was conducted in accordance with strengthening the reporting of observational studies in epidemiology (STROBE) guidelines.

### 2.5 Statistical analysis

To compare the effects of different prepregnancy BMI values on outcomes, this study reported baseline demographic characteristics as frequencies (percentages) or mean values with standard deviations. Differences were assessed using Pearson's *χ*^2^ test for categorical variables and *t*-tests for continuous variables. Both univariate and multivariate logistic analyses were employed for primary and secondary outcomes, estimating odds ratios (ORs) with 95% confidence intervals (CIs), with *p* < 0.05 indicating statistical significance. All analyses were performed using R version 4.2.3. The study utilized five evaluation methods, including unadjusted logistic regression, multivariable logistic regression, propensity score matching (PSM), inverse probability treatment weighting (IPTW), and overlap weighting (OW). The variance inflation factor was used to detect collinearity between variables before subjecting them to multivariate logistic analysis. PSM was applied by calculating the propensity score for each individual based on observed covariates, and then matching treated and control subjects with similar propensity scores to ensure balance in key covariates between the two groups (14). IPTW involved assigning weights to individuals based on their propensity scores, effectively adjusting the sample distribution and reducing sample selection bias (15). OW, which combines the strengths of PSM and IPTW, was used to create a weighted sample that maintains a balanced distribution, thus enhancing the accuracy of causal inference (16). Covariate balance was assessed using standardized mean differences (SMDs), with SMDs <10% indicating good balance. We applied nearest-neighbor matching with a 1:1 ratio and a random-effects model, setting the caliper at a relative coefficient of 0.1 to improve matching precision. R packages such as “MatchIt” and “Tableone” were utilized for conducting analyses and evaluating balance. Loveplot graphs were utilized for visual comparison.

## 3 Results

### 3.1 Baseline characteristics of the study population

**Figure 1:**
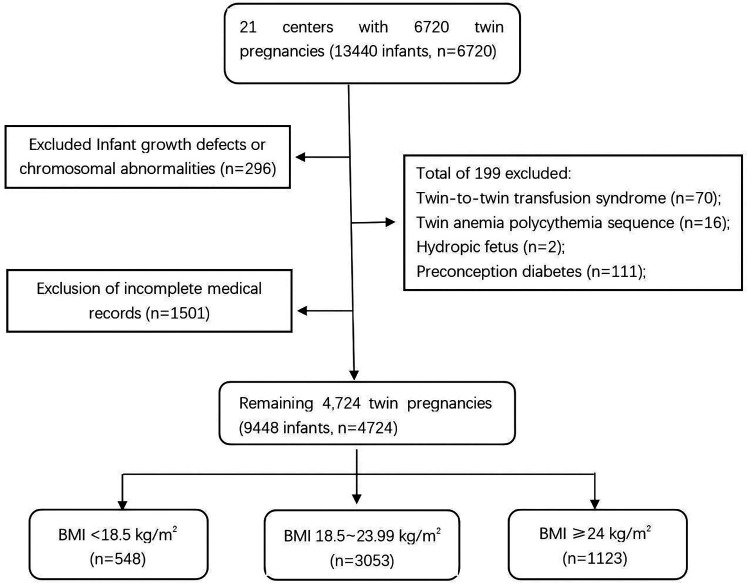
Participant selection process. The flowchart shows the selection of 4,724 twin pregnancies (9,448 infants) from an initial 6,720 pregnancies (13,440 infants). Exclusions included chromosomal abnormalities (*n* = 296), incomplete records (*n* = 1,501), and complications (*n* = 199). Participants were categorized by prepregnancy BMI: < 18.5 kg/m^2^ (*n* = 548), 18.5–23.99 kg/m^2^ (*n* = 3,053), and ≥24 kg/m^2^ (*n* = 1,123).

The dataset initially included 6,720 women with twin pregnancies and clinical data on 14,440 newborns. To reduce confounding factors, cases with chromosomal abnormalities, twin-to-twin transfusion syndrome, twin anemia-polycythemia sequence, hydrops fetalis, or preconception diabetes were excluded. After screening, 4,724 women with twin pregnancies were included in the analysis: 3,053 with normal prepregnancy BMI, 1,123 who were overweight or obese, and 548 who were underweight. The selection process is illustrated in Figure 1.

Table 1 compares the baseline characteristics of women across the BMI categories. Women who were overweight/obese before pregnancy were older, had higher gravidity and parity, and were more likely to conceive through assisted reproduction. This group also had a higher proportion of dichorionic diamniotic twin pregnancies and a higher prevalence of GDM and gestational hypertension. Conversely, they underwent fewer cesarean sections and gained less weight during pregnancy. Similar trends were observed among women with insufficient prepregnancy weight (Supplementary Table S2).

**Table 1:** Baseline characteristics of the unmatched sample, PSM sample, and OW sample for comparison between prepregnancy normal weight and overweight/obesity.

Characteristics	Unmatched sample (*n* = 4,176)		*p* value	PSM (*n* = 2,158)		*p* value	OW (*n* = 1,576)		*p* value
	Normal weight (*n* = 3,053)	Overweight/obesity (*n* = 1,123)		Normal weight (*n* = 1,079)	Overweight/obesity (*n* = 1,079)		Normal weight (*n* = 788)	Overweight/obesity (*n* = 788)	
Advanced maternal age	722 (23.6)	324 (28.9)	0.001	282 (26.1)	299 (27.7)	0.437	212.8 (27.0)	212.8 (27.0)	1.000
Multiparous	1,138 (37.3)	467 (41.6)	0.012	461 (42.7)	440 (40.8)	0.383	318.0 (40.4)	318.0 (40.4)	1.000
Parity			0.005			0.886			1.000
≤1	1,357 (44.4)	440 (39.2)		421 (39.0)	430 (39.9)		320.4 (40.7)	320.4 (40.7)	
2	808 (26.5)	307 (27.3)		294 (27.2)	295 (27.3)		214.4 (27.2)	214.4 (27.2)	
≥3	888 (29.1)	376 (33.5)		364 (33.7)	354 (32.8)		253.1 (32.1)	253.1 (32.1)	
Ethnicity	111 (3.6)	48 (4.3)	0.362	31 (2.9)	44 (4.1)	0.158	31.6 (4.0)	31.6 (4.0)	1.000
Mode of conception	1,914 (62.7)	756 (67.3)	0.006	731 (67.7)	720 (66.7)	0.647	520.3 (66.0)	520.3 (66.0)	1.000
Mode of delivery	2,827 (92.6)	1,018 (90.7)	0.045	991 (91.8)	980 (90.8)	0.444	718.3 (91.2)	718.3 (91.2)	1.000
Pregnancy weight gain	1,509 (49.4)	415 (37.0)	<0.001	411 (38.1)	409 (37.9)	0.965	317.5 (40.3)	317.5 (40.3)	1.000
Adverse pregnancy history	1,297 (42.5)	513 (45.7)	0.067	491 (45.5)	487 (45.1)	0.897	351.5 (44.6)	351.5 (44.6)	1.000
Hypertensive disorder of pregnancy	440 (14.4)	257 (22.9)	<0.001	208 (19.3)	217 (20.1)	0.665	154.1 (19.6)	154.1 (19.6)	1.000
Gestational diabetes	676 (22.1)	353 (31.4)	<0.001	317 (29.4)	316 (29.3)	1.000	223.2 (28.3)	223.2 (28.3)	1.000
Second and third trimester infections	202 (6.6)	59 (5.3)	0.113	44 (4.1)	56 (5.2)	0.26	43.5 (5.5)	43.5 (5.5)	1.000
Abnormal thyroid function	333 (10.9)	137 (12.2)	0.246	128 (11.9)	129 (12.0)	1.000	92.9 (11.8)	92.9 (11.8)	1.000
Anemia	866 (28.4)	286 (25.5)	0.066	260 (24.1)	281 (26.0)	0.321	207.7 (26.4)	207.7 (26.4)	1.000
Chorionicity	2,357 (77.2)	909 (80.9)	0.010	869 (80.5)	871 (80.7)	0.957	628.9 (79.8)	628.9 (79.8)	1.000

PSM, propensity score-matched; OW, overlap weighting.

### 3.2 Primary outcomes

#### 3.2.1 Normal prepregnancy weight vs. overweight/obesity

##### 3.2.1.1 Bivariable analysis

In the unmatched sample of 4,176 twins, those born to mothers who were overweight/obese before pregnancy had a higher incidence of 1-min Apgar scores ≤7 compared to those born to mothers with normal prepregnancy weight (7.7% vs. 5.1% for the larger twin and 7.6% vs. 5.2% for the smaller twin). A similar trend was observed for 5-min Apgar scores ≤7 (1.7% vs. 0.8% for the larger twin and 1.4% vs. 0.8% for the smaller twin) (Model 1).

The crude ORs (95%CIs) from unadjusted logistic regression (Model 1) were 1.57 (1.19–2.06) for the larger twin and 1.49 (1.13–1.95) for the smaller twin in the 1-min Apgar score ≤7 group. After adjusting for confounders using multivariable logistic regression (Model 2), maternal prepregnancy overweight/obesity remained significantly associated with an increased risk of low 1-min Apgar scores, with ORs (95% CIs) of 1.60 (1.20–2.13) for the larger twin and 1.45 (1.09–1.92) for the smaller twin (Table 2).

**Table 2:** The results of OR (95% CIs) for comparisons between prepregnancy normal weight and overweight/obesity in each model.

	Normal weight (*n* = 3,053)	Overweight/Obesity (*n* = 1,123)	OR (95%CIs)				
			Crude	Adjusted	PSM	IPTW	OW
Total (n/%)							
GA <37 weeks	1,909 (62.5)	723 (64.4)	1.08 (0.94–1.25)	0.99 (0.84–1.15)	0.99 (0.82–1.20)	1.00 (0.87–1.13)	0.99 (0.80–1.23)
GA <34 weeks	483 (15.8)	215 (19.1)	1.25 (1.05–1.50)	1.21 (0.99–1.47)	1.19 (0.93–1.52)	1.20 (1.02–1.42)	1.21 (0.91–1.61)
BWDT ≥20%	520 (17.0)	191 (17.0)	1.00 (0.83–1.20)	0.96 (0.80–1.16)	0.92 (0.74–1.15)	0.95 (0.81–1.11)	0.96 (0.74–1.25)
Larger twin							
1 min Apgar ≤7	155 (5.1)	87 (7.7)	1.57 (1.19–2.06)	1.60 (1.20–2.13)	1.60 (1.11–2.33)	1.67 (1.31–2.12)	1.65 (1.08–2.57)
5 min Apgar ≤7	24 (0.8)	16 (1.4)	0.93 (0.27–2.41)	0.82 (0.24–2.17)	0.62 (0.15–2.26)	0.80 (0.26–1.96)	0.82 (0.17–3.71)
NICU admission	1,426 (46.7)	531 (47.3)	1.02 (0.89–1.17)	0.961 (0.83–1.11)	0.95 (0.80–1.14)	0.97 (0.86–1.10)	0.96 (0.78–1.19)
Low birth weight	1,365 (44.7)	468 (41.7)	0.88 (0.77–1.01)	0.80 (0.68–0.92)	0.76 (0.63–0.91)	0.80 (0.71–0.91)	0.95 (0.81–1.11)
Smaller twin							
1 min Apgar ≤7	159 (5.2)	85 (7.6)	1.49 (1.13–1.95)	1.45 (1.09–1.92)	1.55 (1.07–2.25)	1.48 (1.17–1.87)	1.47 (0.97–2.25)
5 min Apgar ≤7	24 (0.8)	15 (1.3)	1.71 (0.87–3.24)	1.40 (0.70–2.73)	0.96 (0.43–2.14)	1.46 (0.84–2.52)	1.42 (0.53–4.02)
NICU admission	1,536 (50.3)	576 (51.3)	1.04 (0.91–1.19)	0.96 (0.83–1.11)	0.96 (0.81–1.15)	0.96 (0.85–1.09)	0.96 (0.78–1.18)
Low birth weight	2,188 (71.7)	770 (68.6)	0.86 (0.74–1.00)	0.78 (0.67–0.91)	0.75 (0.62–0.91)	0.78 (0.68–0.89)	0.78 (0.62–0.98)
Complications of pregnancy							
Hypertensive disorder of pregnancy	440 (14.4)	257 (22.9)	1.71 (1.43–2.03)	1.85 (1.55–2.21)	1.73 (1.38–2.16)	1.87 (1.61–2.18)	1.85 (1.43–2.42)
Gestational diabetes	676 (22.1)	353 (31.4)	1.61 (1.38–1.88)	1.49 (1.27–1.74)	1.47 (1.21–1.78)	1.48 (1.30–1.68)	1.49 (1.19–1.86)
Second and third trimester infections	202 (6.6)	59 (5.3)	0.78 (0.58–1.05)	0.76 (0.56–1.02)	0.68 (0.48–0.96)	0.75 (0.58–0.97)	0.76 (0.50–1.15)
Abnormal thyroid function	333 (10.9)	137 (12.2)	1.13 (0.92–1.40)	1.14 (0.92–1.42)	1.04 (0.81–1.35)	1.15 (0.96–1.38)	1.14 (0.84–1.56)
Anemia	866 (28.4)	286 (25.5)	0.86 (0.74–1.01)	0.83 (0.71–0.97)	0.84 (0.69–1.01)	0.84 (0.73–0.95)	0.83 (0.66–1.03)

BWDT, birth weight discordance in twins; CIs, confidence intervals; GA, gestational age; IPTW, inverse probability treatment weighting; OR, odds ratios; OW, overlap weighting; PSM, propensity score-matched.

However, no significant associations were found for 5-min Apgar scores ≤7. The crude ORs (95% CIs) from unadjusted logistic regression (Model 1) were 0.93 (0.27–2.41) for the larger twin and 1.71 (0.87–3.24) for the smaller twin. After adjustment (Model 2), maternal prepregnancy overweight/obesity showed no significant impact on the risk of low 5-min Apgar scores, with ORs (95% CIs) of 0.82 (0.24–2.17) for the larger twin and 1.40 (0.70–2.73) for the smaller twin (Table 2).

#### 3.2.2 Propensity score-matched analysis

To investigate the impact of maternal prepregnancy BMI on the 1-min and 5 min Apgar score of twin infants, it was essential to eliminate confounding factors such as preeclampsia and GDM, which have been indicated to affect Apgar scores (17, 18). A 1:1 PSM analysis (Model 3) was performed. As illustrated in Figure 2, all baseline variables had SMDs of less than 0.1, indicating that the two groups were well balanced after matching.

The results revealed that twin infants born to mothers with prepregnancy overweight/obesity were at a higher risk of having a 1-min Apgar score ≤7 compared to those born to mothers with normal prepregnancy weight, with ORs (95% CIs) of 1.60 (1.11–2.33) for the larger twin and 1.55 (1.07–2.25) for the smaller twin, respectively (Table 2). In contrast, no significant differences were observed in the 5-min Apgar score ≤7 group, with ORs (95% CIs) of 0.62 (0.15–2.26) for the larger twin and 0.96 (0.43–2.14) for the smaller twin.

#### 3.2.3 Inverse probability treatment weighting or overlap weighting analysis

Weighted multivariable logistic regression analyses using IPTW (Model 4) and OW (Model 5) revealed that maternal prepregnancy overweight/obesity increased the risk of a 1-min Apgar score ≤7 in the larger twin, with ORs (95% CI) of 1.67 (1.31–2.12) and 1.65 (1.08–2.57), respectively (Table 2; Supplementary Table S3). Similar results were observed for smaller twin in the analysis with IPTW, while the analysis with OW almost reached significance, with ORs (95% CI, *p* value) of 1.48 (1.17–1.87, 0.001) and 1.46 (0.97–2.25, 0.07), respectively. In contrast, analyses for 5-min Apgar scores ≤7 confirmed no significant differences using either IPTW or OW (Table 2; Supplementary Table S3). Weighted baseline characteristics were poorly balanced for IPTW but well-balanced for OW (Supplementary Figure S1).

### 3.3 Normal prepregnancy weight vs. underweight

In contrast to the increased risk associated with prepregnancy overweight/obesity, maternal prepregnancy underweight was associated with a decreased risk of 1-min Apgar score ≤7 in twins. For the larger twin, the ORs (95% CIs) across the models were 0.56 (0.32–0.92) in Model 1, 0.50 (0.28–0.83) in Model 2, 0.48 (0.24–0.89) in Model 3, 0.49 (0.29–0.78) in Model 4, and 0.50 (0.24–0.98) in Model 5 (Supplementary Table S4). For the smaller twin, the ORs (95% CIs) values in Models 1–5 were 0.58 (0.34–0.94), 0.56 (0.32–0.91), 0.49 (0.25–0.91), 0.55 (0.33–0.86), and 0.55 (0.27–1.07), respectively (Supplementary Table S4). However, no significant differences were observed for 5-min Apgar scores ≤7 across Models 1–5. The baseline characteristics before and after matching or weighting were balanced, as shown in Supplementary Figure S2.

### 3.4 Other outcomes

Furthermore, the study also examined the impact of different prepregnancy BMI values on various outcomes, including NICU admission, preterm birth (GA <37 weeks), early preterm birth (GA <34 weeks), LBW, and BWDT. Compared to normal prepregnancy BMI, prepregnancy overweight/obesity had a protective effect on smaller twin, reducing the likelihood of LBW. However, maternal normal weight before pregnancy did not have a significant effect on other outcomes, such as NICU admission, preterm birth, early preterm birth, and BWDT, when compared to maternal prepregnancy overweight/obesity or underweight (Table 2; Supplementary Table S4).

### 3.5 Prepregnancy BMI and pregnancy complications

Table 1 shows the association between different BMI values and maternal pregnancy-related diseases. According to the analysis of Models 1–5, compared with normal prepregnancy weight, maternal overweight/obesity before pregnancy not only increased the risk of developing GDM during pregnancy, with ORs (95% CIs) of 1.61 (1.38–1.88), 1.49 (1.27–1.74), 1.47 (1.21–1.78), 1.48 (1.30–1.68), and 1.49 (1.19–1.86), respectively, but also increased the likelihood of developing gestational hypertension, with ORs (95% CIs) of 1.71 (1.43–2.03), 1.85 (1.55–2.21), 1.73 (1.38–2.16), 1.87 (1.61–2.18), and 1.85 (1.43–2.42) (Table 2; Supplementary Table S5). Conversely, prepregnancy underweight did not present such risks (Supplementary Tables S4–S6). Furthermore, different prepregnancy BMI values did not exhibit significant effects on the occurrence of thyroid function abnormalities, anaemia, and infections during mid-late pregnancy.

### 3.6 Stratified analysis and sensitivity analysis

To ensure the reliability of our findings regarding the impact of different prepregnancy BMI categories on 1-min and 5-min Apgar scores, we performed sensitivity analyses by stratifying twins based on GA and BWDT. The results showed that, compared to normal weight, prepregnancy overweight/obesity significantly increased the risk of 1-min Apgar scores ≤7 in twins with a GA less than 34 weeks (Table 3). However, when stratified by BWDT, prepregnancy BMI did not consistently show a significant association with 1-min or 5-min Apgar scores ≤7 (Table 3). Similar results were observed when comparing prepregnancy underweight with normal weight (Supplementary Table S7).

**Table 3:** Summary of sensitivity analysis between prepregnancy normal weight and overweight/obesity.

	Normal weight (*n* = 3,053)	Overweight/Obesity (*n* = 1,123)	OR (95%CIs)				
			Crude	Adjusted	PSM	IPTW	OW
Larger twin (*n*/%)							
GA>37 weeks	11 (1.0)	3 (0.8)	0.78 (0.18–2.51)	0.73 (0.14–2.62)	3.82 (0.49–2.16)	7.85 (0.21–2.37)	0.92 (0.12–6.64)
34–36 weeks	61 (4.3)	20 (3.9)	0.92 (0.53–1.51)	1.09 (0.63–1.83)	0.72 (0.37–1.38)	1.12 (0.70–1.74)	1.12 (0.51–2.45)
GA < 34 weeks	83 (17.2)	64 (29.8)	2.04 (1.40–2.97)	1.94 (1.30–2.89)	1.90 (1.17–3.12)	1.98 (1.43–2.73)	1.94 (1.08–3.53)
BWDT ≥20%	128 (5.1)	76 (8.2)	1.12 (0.52–2.24)	1.16 (0.52–2.42)	0.90 (0.33–2.40)	1.14 (0.59–2.11)	1.22 (0.39–3.86)
BWDT<20%	27 (5.2)	11 (5.8)	1.67 (1.24–2.23)	1.73 (1.26–2.35)	1.87 (1.23–2.87)	1.83 (1.41–2.37)	1.79 (1.12–2.91)
Smaller twin							
GA>37 weeks	16 (1.4)	4 (1.0)	0.71 (0.20–1.96)	0.88 (0.24–2.49)	0.46 (0.11–1.63)	0.85 (0.28–2.17)	0.86 (0.16–4.51)
34–36 weeks	63 (4.4)	18 (3.5)	0.79 (0.45–1.33)	0.89 (0.50–1.51)	0.97 (0.48–1.97)	0.88 (0.55–1.39)	0.89 (0.40–1.94)
GA < 34 weeks	80 (16.6)	63 (29.3)	2.09 (1.43–3.05)	1.97 (1.32–2.93)	1.71 (1.07–2.78)	1.94 (1.41–2.67)	1.97 (1.11–3.57)
BWDT ≥20%	133 (5.3)	76 (8.2)	2.10 (1.12–3.87)	2.34 (1.20–4.47)	2.20 (0.89–5.86)	2.35 (1.35–4.09)	2.34 (0.88–6.84)
BWDT<20%	26 (5.0)	66 (34.6)	1.38 (1.01–1.86)	1.30 (0.94–1.78)	1.15 (0.77–1.71)	1.34 (1.02–1.74)	1.32 (0.82–2.12)

BWDT, birth weight discordance in twins; CIs, confidence intervals; GA, gestational age; IPTW, inverse probability treatment weighting; OR, odds ratios; OW, overlap weighting; PSM, propensity score-matched.

In addition, we conducted subgroup analyses for overweight and obesity. We found that prepregnancy overweight increased the risk of 1-min Apgar scores ≤7 for both larger twins (adjusted OR 1.60, 95% CI: 1.17–2.18) and smaller twins (adjusted OR 1.43, 95% CI: 1.04–1.94). Prepregnancy obesity, however, was associated with an increased risk of 1-min Apgar scores ≤7 only in larger twins (adjusted OR 1.75, 95% CI: 1.03–2.86) but not in smaller twins (adjusted OR 1.58, 95% CI: 0.94–2.54). Additionally, validation methods such as PSM or OW failed to yield consistent results (Supplementary Table S7, S8).

Further sensitivity analyses were performed by adjusting the matching algorithm using various caliper values (0.02, 0.1, 0.2) and matching ratios (1:1, 2:1) or applying a probit model. Regardless of these adjustments, the statistical significance of the results remained consistent, confirming the robustness of our effect estimates (data not shown).

## 4 Discussion

Our study revealed that maternal prepregnancy overweight/obesity increases the risk of adverse outcomes in twin pregnancies, particularly a higher risk of a 1-min Apgar score ≤7 in both larger and smaller twins, while no significant association was observed with 5-min Apgar scores. Sensitivity analysis further indicated that this effect is primarily evident in twins with a GA of less than 34 weeks. Interestingly, compared to normal prepregnancy weight, maternal underweight was associated with a lower risk of 1-min Apgar scores ≤7, suggesting a progressive increase in risk with rising prepregnancy BMI. Additionally, secondary findings demonstrated that prepregnancy overweight/obesity is associated with a higher risk of gestational hypertension and GDM, emphasizing the importance of maternal weight management before pregnancy.

Our analysis consistently showed that prepregnancy overweight or obesity is associated with adverse outcomes across various models, even after adjusting for multiple prepregnancy covariates. Specifically, our findings align with previous research indicating that, in twin pregnancies, prepregnancy overweight increases the risk of GDM, HDP, and low Apgar scores (8, 19–23). Studies that do not distinguish between singleton and twin pregnancies similarly report that higher prepregnancy BMI is associated with increased risks of GDM, neonatal mortality, preeclampsia, and low Apgar scores (24, 25). Comparable findings have also been observed in singleton pregnancies, where elevated prepregnancy BMI is linked to lower Apgar scores (26). These results suggest that although twin pregnancies are generally at a higher risk for adverse outcomes, prepregnancy overweight or obesity contributes similarly to adverse outcomes in both singleton and twin pregnancies.

The prevalence of obesity is increasing across all age groups and social strata due to environmental and genetic factors (27, 28). While the exact mechanisms by which prepregnancy obesity contributes to low Apgar scores in newborns remain unclear, several factors are generally recognized. Firstly, obesity induces metabolic disorders, such as inflammation and abnormal blood sugar and lipid levels, which may cross the placenta and negatively impact fetal health (29, 30). Additionally, obesity can lead to structural and functional changes in the placenta, potentially restricting fetal nutrition and oxygen supply, which increases the risk of low Apgar scores (3, 31). Moreover, prepregnancy obesity is frequently associated with conditions like hypertension and diabetes, further compounding this risk (18, 32, 33).

Twin pregnancies also introduce additional metabolic challenges, such as vitamin D deficiency and elevated bile acid levels. Vitamin D deficiency, which is more common in twin pregnancies due to the increased nutritional demands, has been associated with impaired fetal development and lower Apgar scores (34, 35). Elevated bile acid levels, often seen in twin pregnancies, can lead to hepatic dysfunction and impaired fetal circulation, further increasing the risk of adverse neonatal outcomes (36, 37). Both of these factors have been linked to lower Apgar scores, which reflect the newborn's immediate post-birth health and response to environmental stress. Finally, the combination of obesity and twin pregnancy substantially increases the complexity of cesarean sections, often resulting in longer surgery and anesthesia times, along with heightened risks of infection and fetal distress—all of which may contribute to lower Apgar scores in newborns (38–40). In summary, prepregnancy obesity and the metabolic challenges associated with twin pregnancies can collectively impact newborn outcomes through multiple pathways.

Our study highlights a significant association between maternal prepregnancy overweight/obesity and low 1-min Apgar scores in twins, particularly those born at gestational ages <34 weeks, while no such association was observed for 5-min Apgar scores. This contrast underscores the immediate impact of maternal obesity-related factors, such as impaired uteroplacental perfusion and fetal hypoxia, which contribute to neonatal distress shortly after birth (41, 42). However, the absence of an association with 5-min Apgar scores suggests that timely and effective perinatal interventions, including resuscitation, oxygen supplementation, and other neonatal care measures, are crucial in mitigating these challenges and facilitating neonatal recovery and stabilization within the first 5 min after birth (43–45).

The differences observed between 1-min and 5-min Apgar scores carry important clinical implications. The association between maternal prepregnancy obesity/overweight and low 1-min Apgar scores highlights the role of obesity-related comorbidities, such as gestational hypertension and diabetes, in impairing uteroplacental perfusion, leading to fetal hypoxia and neonatal distress immediately after birth (5, 46). The lack of association with 5-min Apgar scores underscores the effectiveness of prompt perinatal interventions in alleviating these risks (47). Obesity-related inflammation, oxidative stress, and metabolic disturbances likely exacerbate risks of suboptimal uteroplacental perfusion and fetal oxygenation, increasing the likelihood of low 1-min Apgar scores (48, 49), while rapid stabilization through medical care may explain the absence of a 5-min association (50). Despite the observational nature of this study and potential residual confounding, robust sensitivity analyses strengthen the credibility of these findings. Clinically, these results emphasize the importance of optimizing maternal BMI before pregnancy and implementing targeted interventions for at-risk mothers, particularly those with preterm twins, to mitigate immediate postpartum risks and improve neonatal outcomes.

Interestingly, maternal underweight was associated with a lower risk of low 1-min Apgar scores, suggesting a potential inverse relationship between BMI and neonatal outcomes. This may reflect differences in placental structure or metabolic adaptations across BMI categories, as underweight mothers might experience more efficient placental function, optimizing nutrient and oxygen transfer to the fetus and reducing the risk of immediate neonatal distress (51, 52). Additionally, lower maternal adiposity could minimize inflammatory processes and metabolic disturbances commonly seen in overweight and obese individuals, further contributing to favorable outcomes (53). However, maternal underweight also poses significant risks during pregnancy, including inadequate nutritional reserves, which can lead to fetal growth restriction, low birth weight, and preterm birth, along with potential long-term developmental challenges for the neonate (54, 55). Underweight mothers are also at higher risk of complications such as anemia, reduced immune function, and overall poor maternal health during and after pregnancy (56, 57). These findings emphasize the importance of achieving a balanced and healthy BMI prior to pregnancy to optimize outcomes for both mother and child.

The dangers of low Apgar scores are widely recognized. If left untreated, they can lead to neonatal asphyxia and ischaemic hypoxic encephalopathy, which can adversely affect newborn intellectual development and place significant burdens on families and society. Therefore, it is crucial to identify high-risk factors that may contribute to low Apgar scores in advance. In addition to prepregnancy overweight/obesity, as found in this study, other factors, such as excessive weight gain during pregnancy, infections during pregnancy, and placental abruption, can lead to low Apgar scores (58–60). This article provides evidence of the impact of prepregnancy BMI on the Apgar scores of twins, emphasizing the importance of prepregnancy weight management and preparedness for neonatal resuscitation.

Our study has several strengths (1): To our knowledge, it is one of the few studies in China to investigate the impact of prepregnancy BMI on adverse outcomes such as low Apgar scores in twins; (2) The study included multiple medical units from several provincial administrative regions in China, providing a representative and timely sample; (3) The use of PSM, IPTW, and OW can simulate a randomized controlled trial, thereby reducing the risk of indication bias (61, 62). These techniques are crucial in addressing and reducing the risk of indication bias, which can arise due to non-random assignment of treatment groups in observational studies. By mimicking the conditions of a RCT, we have enhanced the robustness of our findings and provided more reliable estimates of the effect of prepregnancy BMI on twin outcomes. (4) Through strict methods, including five different models and three sensitivity analyses, we obtained consistent results among twin foetuses, and the stability of the results is relatively good.

We acknowledge that our study has several limitations. The inclusion of additional covariates in our analysis led to the exclusion of some data. Nevertheless, the consistency of our results across all models suggests that our findings are robust. Moreover, we combined overweight and obesity into a single group to enhance statistical power. While this approach is supported by the similarity in their mechanisms, it may introduce confounding bias and dilution effects due to differences in the degree of impact associated with these weight categories. Additionally, the relatively small sample size of our study may limit the generalizability of our findings, and future research should aim to incorporate larger-scale datasets to validate these results. Finally, as our study was retrospective in nature, we were unable to control for all potential confounding factors. Despite these limitations, our study provides valuable insights into the impact of prepregnancy BMI on neonatal outcomes in twin pregnancies and underscores the importance of further research in this area.

## 5 Conclusion

Prepregnancy overweight/obesity is associated with adverse outcomes, including a higher risk of low Apgar scores in twins, as well as HDP and GDM in twin pregnancies. These findings underscore the importance of guiding prepregnancy weight management and advising paediatricians to prepare for the prevention and treatment of neonatal asphyxia caused by low Apgar scores in twins.

## Data Availability

The raw data supporting the conclusions of this article will be made available by the authors, without undue reservation.

## Supplementary material

Supplementary Figure S1 Love plot of baseline variable balance between normal weight and overweight/obesity groups. This Love plot visualizes the standardized mean difference (SMD) values for baseline variables between normal weight and overweight/obesity groups in unmatched and adjusted models, including propensity score matching (PSM), inverse probability treatment weighting (IPTW), and overlap weighting (OW). Variables assessed include prepregnancy BMI, pregnancy weight gain, hypertensive disorder, gestational diabetes, advanced maternal age, parity, mode of conception, chorionicity, multiparous status, mode of delivery, anemia, adverse pregnancy history, infections in the second and third trimesters, abnormal thyroid function, and ethnicity. An SMD <10% indicates a relatively good balance between groups.

Supplementary Figure S2 Love plot of baseline variable balance between underweight and normal weight groups. This Love plot depicts the standardized mean difference (SMD) values for baseline variables between underweight and normal weight groups across different models, including the unmatched model and adjusted models: propensity score matching (PSM), inverse probability treatment weighting (IPTW), and overlap weighting (OW). The key variables assessed are prepregnancy BMI, advanced maternal age, mode of conception, gestational diabetes, ethnicity, hypertensive disorders, chorionicity, parity, abnormal thyroid function, multiparity, adverse pregnancy history, pregnancy weight gain, second- and third-trimester infections, mode of delivery, and anemia. An SMD of less than 10% signifies a relatively good balance between the groups. This analysis demonstrates the effectiveness of these models in achieving covariate balance for more reliable comparisons.
