# Supplementary material for: Maternal prepregnancy overweight/obesity increase the risk of low Apgar scores in twins: a population-based cohort study in China
Source: Front Pediatr. 2025 Jan 17;12:1412975. doi: 10.3389/fped.2024.1412975 (PMC11782157; doi:10.3389/fped.2024.1412975)
Supplement: Supplementary file 1 [file Table1.docx]

**Table S1. The definitions and categories about the covariates**

| **Covariates** | **Categories*** |
| --- | --- |
| Advanced maternal age | No; Yes |
| Multiparous | No; Yes |
| Parity | ≤1; 2; ≥3 |
| Ethnicity | Han; Minority nationality |
| Mode of conception | Nature conceived; No |
| Mode of delivery | Vaginal delivery; Cesarean section |
| Pregnancy weight gain | <0.46 kg/weeks; ≥0.46 kg/weeks |
| Adverse pregnancy history | No; Yes |
| Hypertensive disorder of pregnancy | No; Yes |
| Gestational diabetes | No; Yes |
| Second and third trimester infections | No; Yes |
| Abnormal thyroid function | No; Yes |
| Anemia  Chorionicity | No; Yes  Dichorionic diamniotic twin pregnancy; Monochorionic diamniotic twin pregnancy |

* All are categorical variable

**Table S2. Baseline characteristics of the unmatched sample, PSM sample, and OW sample for comparison between prepregnancy normal weight and underweight.**

| **Characteristics** | **Unmatched Sample (n=3601)** | | **p value** | **PSM (n=1088)** | | **p value** | **OW (n=910)** | | **p value** |
| --- | --- | --- | --- | --- | --- | --- | --- | --- | --- |
|  | **Normal weight (n=3053)** | **Underweight (n=****548)** |  | **Normal weight (n=544)** | **Underweight (n=544)** |  | **Normal weight (n=455)** | **Underweight**  **(n=455)** |  |
| Advanced maternal age | 722 (23.6) | 76 (13.9) | <0.001 | 75 (13.8) | 76 (14.0) | 1 | 68.3 (15.0) | 68.3 (15.0) | 1 |
| Multiparous | 1,138 (37.3) | 183 (33.4) | 0.083 | 181 (33.3) | 183 (33.6) | 0.949 | 154.2 (33.9) | 154.2 (33.9) | 1 |
| Parity |  |  | 0.073 |  |  | 0.942 |  |  | 1 |
| ≤1 | 1,357 (44.4) | 271 (49.5) |  | 272 (50.0) | 269 (49.4) |  | 221.3 (48.6) | 221.3 (48.6) |  |
| 2 | 808 (26.5) | 139 (25.4) |  | 132 (24.3) | 137 (25.2) |  | 117.1 (25.7) | 117.1 (25.7) |  |
| ≥3 | 888 (29.1) | 138 (25.2) |  | 140 (25.7) | 138 (25.4) |  | 116.9 (25.7) | 116.9 (25.7) |  |
| Ethnicity | 111 (3.6) | 9 (1.6) | 0.014 | 5 (0.9) | 9 (1.7) | 0.421 | 8.2 (1.8) | 8.2 (1.8) | 1 |
| Mode of conception | 1,914 (62.7) | 289 (52.7) | <0.001 | 298 (54.8) | 289 (53.1) | 0.627 | 247.3 (54.3) | 247.3 (54.3) | 1 |
| Mode of delivery | 2,827 (92.6) | 502 (91.6) | 0.429 | 511 (93.9) | 500 (91.9) | 0.237 | 417.0 (91.6) | 417.0 (91.6) | 1 |
| Pregnancy weight gain | 1,509 (49.4) | 291 (53.1) | 0.115 | 297 (54.6) | 288 (52.9) | 0.627 | 238.7 (52.4) | 238.7 (52.4) | 1 |
| Adverse pregnancy history | 1,297 (42.5) | 211 (38.5) | 0.090 | 206 (37.9) | 209 (38.4) | 0.901 | 178.3 (39.2) | 178.3 (39.2) | 1 |
| Hypertensive disorder of pregnancy | 440 (14.4) | 57 (10.4) | 0.013 | 60 (11.0) | 57 (10.5) | 0.845 | 49.7 (10.9) | 49.7 (10.9) | 1 |
| Gestational diabetes | 676 (22.1) | 84 (15.3) | <0.001 | 98 (18.0) | 84 (15.4) | 0.291 | 73.3 (16.1) | 73.3 (16.1) | 1 |
| Second and third trimester infections | 202 (6.6) | 28 (5.1) | 0.217 | 18 (3.3) | 28 (5.1) | 0.175 | 24.1 (5.3) | 24.1 (5.3) | 1 |
| Abnormal thyroid function | 333 (10.9) | 46 (8.4) | 0.082 | 39 (7.2) | 46 (8.5) | 0.498 | 40.3 (8.8) | 40.3 (8.8) | 1 |
| Anemia | 866 (28.4) | 164 (29.9) | 0.472 | 155 (28.5) | 162 (29.8) | 0.689 | 135.1 (29.7) | 135.1 (29.7) | 1 |
| Chorionicity | 2,357 (77.2) | 396 (72.3) | 0.014 | 395 (72.6) | 394 (72.4) | 1 | 333.2 (73.2) | - 1. (73.2) | 1 |

PSM, propensity score-matched; OW, overlap weighting.

**Table S3. Baseline characteristics of the IPTW sample for prepregnancy BMI comparisons.**

| **Characteristics** | **Normal weight**  **(n= 4174)** | **Overweight/**  **obesity (n= 1638)** | | **p value** | **Normal weight**  **(n=3601)** | **Underweight**  **(n=662)** | **p value** |
| --- | --- | --- | --- | --- | --- | --- | --- |
| Advanced maternal age | 1043.2 (25.0) | | 507.8 (31.0) | <0.001 | 798.2 (22.2) | 84.6 (12.8) | <0.001 |
| Multiparous | 1606.1 (38.5) | | 704.1 (43.0) | 0.009 | 1320.5 (36.7) | 217.8 (32.9) | 0.091 |
| Parity |  | |  | 0.003 |  |  | 0.067 |
| ≤1 | 1795.6 (43.0) | | 615.1 (37.6) |  | 1627.2 (45.2) | 333.1 (50.3) |  |
| 2 | 1115.0 (26.7) | | 447.8 (27.3) |  | 948.0 (26.3) | 165.5 (25.0) |  |
| ≥3 | 1263.8 (30.3) | | 574.6 (35.1) |  | 1025.8 (28.5) | 163.4 (24.7) |  |
| Ethnicity | 158.1 (3.8) | | 74.7 (4.6) | 0.278 | 120.0 (3.3) | 9.8 (1.5) | 0.015 |
| Mode of conception | 2669.0 (63.9) | | 1125.5 (68.7) | 0.004 | 2202.7 (61.2) | 338.3 (51.1) | <0.001 |
| Mode of delivery | 3842.6 (92.1) | | 1475.0 (90.1) | 0.052 | 3327.9 (92.4) | 606.4 (91.6) | 0.511 |
| Pregnancy weight gain | 1924.7 (46.1) | | 548.0 (33.5) | <0.001 | 1799.4 (50.0) | 356.2 (53.8) | 0.098 |
| Adverse pregnancy history | 1807.8 (43.3) | | 766.7 (46.8) | 0.045 | 1508.6 (41.9) | 250.4 (37.8) | 0.074 |
| Hypertensive disorder of pregnancy | 691.2 (16.6) | | 442.1 (27.0) | <0.001 | 497.0 (13.8) | 65.5 (9.9) | 0.011 |
| Gestational diabetes | 1028.4 (24.6) | | 574.7 (35.1) | <0.001 | 759.6 (21.1) | 96.4 (14.6) | <0.001 |
| Second and third trimester infections | 260.0 (6.2) | | 81.5 (5.0) | 0.122 | 229.9 (6.4) | 32.7 (4.9) | 0.188 |
| Abnormal thyroid function | 469.3 (11.2) | | 207.0 (12.6) | 0.223 | 379.6 (10.5) | 52.6 (7.9) | 0.059 |
| Anemia | 1153.6 (27.6) | | 400.2 (24.4) | 0.038 | 1030.1 (28.6) | 199.9 (30.2) | 0.45 |
| Chorionicity | 3262.8 (78.2) | | 1345.1 (82.1) | 0.004 | 2753.5 (76.5) | 471.9 (71.3) | 0.01 |

IPTW, inverse probability treatment weighting.

**Table S4. The results of OR (95% CIs) for comparisons between prepregnancy normal weight and underweight in each model.**

|  | **OR (95%CIs)** | | | | |
| --- | --- | --- | --- | --- | --- |
|  | **Crude** | **Adjusted** | **PSM** | **IPTW** | **OW** |
| **Total** |  |  |  |  |  |
| GA＜37 weeks | 0.82 (0.68-0.99) | 0.82 (0.67-1.00) | 0.69 (0.53-0.90) | 0.82 (0.69-0.99) | 0.82 (0.62-1.09) |
| GA <34 weeks | 0.91 (0.70-1.17) | 0.86 (0.65-1.13) | 0.69 (0.48-0.98) | 0.85 (0.66-1.09) | 0.86 (0.58-1.26) |
| BWDT | 0.92 (0.71-1.17) | 0.94 (0.72-1.20) | 0.73 (0.53-0.99) | 0.95 (0.75-1.19) | 0.93 (0.65-1.33) |
| **Larger twin** |  |  |  |  |  |
| 1 min Apgar ≤7 | 0.56 (0.32-0.92) | 0.50 (0.28-0.83) | 0.48 (0.24-0.89) | 0.49 (0.29-0.78) | 0.50 (0.24-0.98) |
| 5 min Apgar ≤7 | 0.93 (0.27-2.41) | 0.82 (0.24-2.17) | 0.62 (0.15-2.26) | 0.80 (0.26-1.96) | 0.82 (0.17-3.71) |
| NICU admission | 0.90 (0.75-1.08) | 0.90 (0.74-1.10) | 0.75 (0.58-0.96) | 0.90 (0.75-1.07) | 0.90 (0.69-1.19) |
| Low Birth weight | 1.21 (1.01-1.45) | 1.19 (0.98-1.45) | 0.99 (0.76-1.28) | 1.19 (0.99-1.42) | 1.19 (0.90-1.57) |
| **Smaller twin** |  |  |  |  |  |
| 1 min Apgar ≤7 | 0.58 (0.34-0.94) | 0.56 (0.32-0.91) | 0.49 (0.25-0.91) | 0.55 (0.33-0.86) | 0.55 (0.27-1.07) |
| 5 min Apgar ≤7 | 0.23 (0.01-1.09) | 0.22 (0.01-1.08) | 0.26 (0.01-2.20) | 0.22 (0.02-0.96) | 0.23 (0.01-1.77) |
| NICU admission | 0.92 (0.77-1.11) | 0.93 (0.76-1.12) | 0.84 (0.65-1.08) | 0.92 (0.77-1.10) | 0.93 (0.70-1.22) |
| Low Birth weight | 1.22 (0.99-1.51) | 1.24 (1.00-1.55) | 1.08 (0.81-1.44) | 1.24 (1.02-1.52) | 1.24 (0.91-1.69) |
|  |  |  |  |  |  |
| **Complications of pregnancy** |  |  |  |  |  |
| Hypertensive disorder of pregnancy | 0.69 (0.51-0.92) | 0.69 (0.51-0.92) | 0.74 (0.51-1.08) | 0.69 (0.52-0.90) | 0.69 (0.46-1.03) |
| Gestational diabetes | 0.64 (0.49-0.81) | 0.71 (0.55-0.90) | 0.76 (0.55-1.04) | 0.71 (0.56-0.89) | 0.71 (0.50-1.00) |
| Second and third trimester infections | 0.76 (0.50-1.12) | 0.77 (0.50-1.14) | 1.19 (0.68-2.11) | 0.77 (0.52-1.10) | 0.77 (0.43-1.33) |
| Abnormal thyroid function | 0.75 (0.54-1.02) | 0.74 (0.53-1.01) | 0.75 (0.50-1.12) | 0.73 (0.54-0.97) | 0.74 (0.48-1.15) |
| Anemia | 1.08 (0.88-1.31) | 1.10 (0.90-1.34) | 1.10 (0.84-1.43) | 1.10 (0.91-1.32) | - 1. (0.82-1.46) |

BWDT, birth weight discordance in twins; CIs, confidence intervals; GA, gestational age; IPTW, inverse probability treatment weighting; OR, odds ratios; OW, overlap weighting; PSM, propensity score-matched.

**Table S5. Baseline characteristics of the PSM sample, IPTW sample and OW sample for comparison between prepregnancy normal weight and overweight/obesity.**

| **Characteristics** | **PSM (n=2216)** | | **p value** | **SMD** | **IPTW (n=5753)** | | **p value** | **SMD** | **OW (n=1610)** | | **p value** | **SMD** |
| --- | --- | --- | --- | --- | --- | --- | --- | --- | --- | --- | --- | --- |
|  | **Normal weight**  **(n=1,108)** | **Overweight/obesity (n=1,108)** |  |  | **Normal weight**  **(n=4176)** | **Overweight/obesity (n=1577)** |  |  | **Normal weight**  **(n=805)** | **Overweight/obesity (n=805)** |  |  |
| Advanced maternal age | 314 (28.3) | 314 (28.3) | 1 | <0.001 | 1604.1 (38.4) | 677.9 (43.0) | 0.008 | 0.093 | 219.0 (27.2) | 219.0 (27.2) | 1 | <0.001 |
| Multiparous | 461 (41.6) | 459 (41.4) | 0.966 | 0.004 |  |  | 0.003 | 0.12 | 324.2 (40.3) | 324.2 (40.3) | 1 | <0.001 |
| Parity |  |  | 0.994 | 0.006 | 1799.0 (43.1) | 592.2 (37.5) |  |  |  |  | 1 | <0.001 |
| ≤1 | 437 (39.4) | 440 (39.7) |  |  | 1112.7 (26.6) | 435.8 (27.6) |  |  | 328.5 (40.8) | 328.5 (40.8) |  |  |
| 2 | 303 (27.3) | 301 (27.2) |  |  | 1264.0 (30.3) | 549.5 (34.8) |  |  | 217.5 (27.0) | 217.5 (27.0) |  |  |
| ≥3 | 368 (33.2) | 367 (33.1) |  |  | 158.4 (3.8) | 71.2 (4.5) |  |  | 259.3 (32.2) | 259.3 (32.2) |  |  |
| Ethnicity | 39 (3.5) | 41 (3.7) | 0.909 | 0.01 | 2670.5 (64.0) | 1080.2 (68.5) | 0.006 | 0.096 | 32.6 (4.0) | 32.6 (4.0) | 1 | <0.001 |
| Mode of conception | 753 (68.0) | 751 (67.8) | 0.964 | 0.004 | 1923.9 (46.1) | 533.2 (33.8) | <0.001 | 0.253 | 532.8 (66.2) | 532.8 (66.2) | 1 | <0.001 |
| Mode of delivery | 413 (37.3) | 411 (37.1) | 0.965 | 0.004 | 1806.1 (43.3) | 738.9 (46.8) | 0.04 | 0.072 | 323.6 (40.2) | 323.6 (40.2) | 1 | <0.001 |
| Pregnancy weight gain | 501 (45.2) | 503 (45.4) | 0.966 | 0.004 | 3266.0 (78.2) | 1291.5 (81.9) | 0.009 | 0.091 | 358.7 (44.5) | 358.7 (44.5) | 1 | <0.001 |
| Adverse pregnancy history | 900 (81.2) | 899 (81.1) | 1 | 0.002 | 1604.1 (38.4) | 677.9 (43.0) | 0.008 | 0.093 | 644.4 (80.0) | 644.4 (80.0) | 1 | <0.001 |

IPTW, inverse probability treatment weighting; OW, overlap weighting; PSM, propensity score-matched; SMD, standardized mean difference.

**Table S6. Baseline characteristics of the PSM sample, IPTW sample and OW sample for comparison between prepregnancy normal weight and underweight.**

| **Characteristics** | **PSM (n=1094)** | | **p value** | **SMD** | **IPTW (n=4259)** | | **p value** | **SMD** | **OW (n=916)** | | **p value** | **SMD** |
| --- | --- | --- | --- | --- | --- | --- | --- | --- | --- | --- | --- | --- |
|  | **Normal weight**  **(n=547)** | **Underweight**  **(n=547)** |  |  | **Normal weight**  **(n=3601)** | **Underweight**  **(n=657.8)** |  |  | **Normal weight**  **(n=458)** | **Underweight**  **(n=458)** |  |  |
| Advanced maternal age | 74 (13.5) | 75 (13.7) | 1 | 0.005 | 797.9 (22.2) | 84.6 (12.9) | <0.001 | 0.247 | 68.3 (14.9) | 68.3 (14.9) | 1 | <0.001 |
| Multiparous | 181 (33.1) | 182 (33.3) | 1 | 0.004 | 1320.8 (36.7) | 216.3 (32.9) | 0.088 | 0.08 | 155.1 (33.9) | 155.1 (33.9) | 1 | <0.001 |
| Parity |  |  | 0.99 | 0.009 |  |  | 0.067 | 0.108 |  |  | 1 | <0.001 |
| ≤1 | 273 (49.9) | 271 (49.5) |  |  | 1627.3 (45.2) | 330.7 (50.3) |  |  | 222.6 (48.6) | 222.6 (48.6) |  |  |
| 2 | 136 (24.9) | 138 (25.2) |  |  | 947.5 (26.3) | 165.0 (25.1) |  |  | 117.4 (25.6) | 117.4 (25.6) |  |  |
| ≥3 | 138 (25.2) | 138 (25.2) |  |  | 1026.2 (28.5) | 162.0 (24.6) |  |  | 117.8 (25.7) | 117.8 (25.7) |  |  |
| Ethnicity | 9 (1.6) | 9 (1.6) | 1 | <0.001 | 120.0 (3.3) | 9.8 (1.5) | 0.015 | 0.121 | 8.3 (1.8) | 8.3 (1.8) | 1 | <0.001 |
| Mode of conception | 291 (53.2) | 289 (52.8) | 0.952 | 0.007 | 2202.9 (61.2) | 336.1 (51.1) | <0.001 | 0.204 | 248.7 (54.3) | 248.7 (54.3) | 1 | <0.001 |
| Mode of delivery | 291 (53.2) | 291 (53.2) | 1 | <0.001 | 1799.5 (50.0) | 353.7 (53.8) | 0.102 | 0.076 | 240.1 (52.5) | 240.1 (52.5) | 1 | <0.001 |
| Pregnancy weight gain | 212 (38.8) | 210 (38.4) | 0.95 | 0.008 | 1508.6 (41.9) | 248.8 (37.8) | 0.074 | 0.083 | 179.3 (39.2) | 179.3 (39.2) | 1 | <0.001 |
| Adverse pregnancy history | 393 (71.8) | 395 (72.2) | 0.946 | 0.008 | 2753.3 (76.5) | 469.2 (71.3) | 0.011 | 0.117 | 334.9 (73.2) | 334.9 (73.2) | 1 | <0.001 |

IPTW, inverse probability treatment weighting; OW, overlap weighting; PSM, propensity score-matched; SMD, standardized mean difference.

**Table S7. Summary of Sensitivity Analysis between prepregnancy normal weight and underweight.**

|  | **OR (95%CIs)** | | | | |
| --- | --- | --- | --- | --- | --- |
|  | **Crude** | **Adjusted** | **PSM** | **IPTW** | **OW** |
| **Larger twin** |  |  |  |  |  |
| GA＞37 weeks | 0.90(0.14-3.38) | 0.66(0.09-2.76) | 0.32(0.02-2.91) | 0.66(0.12-2.37) | 0.65(0.04-7.05) |
| 34-36 weeks | 0.78(0.34-1.56) | 0.47(0.16-1.06) | 0.57(0.21-1.46) | 0.71(0.33-1.36) | 0.69(0.23-1.95) |
| GA<34 weeks | 0.39(0.15-0.86) | 0.35(0.13-0.79) | 0.82(0.20-3.25) | 0.34(0.13-0.73) | 0.33(0.09-1.01) |
| BWDT≥20% | 0.92(0.27-2.42) | 0.63(0.18-1.77) | 0.31(0.02-2.86) | 0.63(0.21-1.56) | 0.59(0.10-2.86) |
| BWDT＜20% | 0.54(0.29-0.94) | 0.50(0.27-0.88) | 0.43(0.21-0.86) | 0.49(0.28-0.82) | 0.50(0.22-1.06) |
| **Smaller twin** |  |  |  |  |  |
| GA＞37 weeks | 0.62(0.10-2.18) | 0.53(0.08-2.01) | 0.30(0.04-1.63) | 0.51(0.10-1.73) | 0.52(0.05-3.91) |
| 34-36 weeks | 0.47(0.16-1.06) | 0.45(0.16-1.04) | 0.81(0.22- 2.84) | 0.45(0.17-0.97) | 0.43(0.11-1.38) |
| GA<34 weeks | 0.72(0.34-1.40) | 0.73(0.34-1.44) | 0.59(0.18-1.76) | 0.74(0.36-1.37) | 0.72(0.26-1.97) |
| BWDT≥20% | 0.65(0.15-1.90) | 0.67(0.15-2.08) | 2.03(0.19-44.08) | 0.67(0.18-1.86) | 0.67(0.10-3.91) |
| BWDT＜20% | 0.52(0.28-0.90) | 0.53(0.28-0.92) | 0.55(0.26-1.11) | 0.52(0.29-0.86) | 0.52(0.23-1.10) |

IPTW, inverse probability treatment weighting; OW, overlap weighting; PSM, propensity score-matched.

**Table S8. The results of OR (95% CIs) for comparisons between prepregnancy normal weight and obesity in each model.**

|  | **Normal weight (n=3053)** | **Obesity**  **(n=246)** | **OR (95%CIs)** | | | | |
| --- | --- | --- | --- | --- | --- | --- | --- |
|  |  |  | **Crude** | **Adjusted** | **PSM** | **IPTW** | **OW** |
| **Total** (n/%) |  |  |  |  |  |  |  |
| GA <37 weeks | 1909 (62.5) | 164 (66.7) | 1.20 (0.91-1.58) | 1.04 (0.78-1.41) | 0.95 (0.63-1.44) | 1.04 (0.79-1.39) | 1.04 (0.68-1.59) |
| GA <34 weeks | 483 (15.8) | 40 (16.3) | 1.03 (0.72-1.45) | 0.97 (0.65-1.41) | 1.03 (0.59-1.8) | 0.99 (0.68-1.41) | 0.99 (0.57-1.72) |
| BWDT ≥20% | 520 (17.0) | 43 (17.5) | 1.03 (0.72-1.44) | 0.98 (0.68-1.38) | 0.88 (0.55-1.41) | 0.96 (0.69-1.33) | 0.96 (0.59-1.57) |
| **Larger twin** |  |  |  |  |  |  |  |
| 1 min Apgar ≤ 7 | 155 (5.1) | 20 (8.1) | 1.65 (0.99-2.63) | 1.75 (1.03-2.86) | 1.56 (0.72-3.5) | 1.84 (1.12-2.9) | 1.81 (0.81-4.29) |
| 5 min Apgar ≤ 7 | 24 (0.8) | 4 (1.6) | 2.09 (0.61-5.45) | 2.28 (0.65-6.29) | 1.18 (0.24-6.45) | 2.26 (0.68-5.99) | 2.25 (0.37-21.54) |
| NICU admission | 1426 (46.7) | 119 (48.4) | 1.07 (0.82-1.39) | 0.99 (0.75-1.3) | 0.86 (0.59-1.26) | 1.01 (0.78-1.32) | 0.99 (0.67-1.46) |
| Low Birth weight | 1365 (44.7) | 107 (43.5) | 0.95 (0.73-1.24) | 0.84 (0.63-1.11) | 0.81 (0.55-1.2) | 0.86 (0.65-1.12) | 0.83 (0.56-1.24) |
| **Smaller twin** |  |  |  |  |  |  |  |
| 1 min Apgar ≤ 7 | 159 (5.2) | 21 (8.5) | 1.70 (1.03-2.67) | 1.58 (0.94-2.54) | 0.91 (0.47-1.77) | 1.65 (1.02-2.57) | 1.65 (0.76-3.71) |
| 5 min Apgar ≤ 7 | 24 (0.8) | 2 (0.8) | 1.03 (0.17-3.51) | 0.72 (0.11-2.58) | 0.18 (0.02-0.84) | 0.77 (0.15-2.49) | 0.76 (0.07-6.46) |
| NICU admission | 1536 (50.3) | 122 (49.6) | 1.00 (0.77-1.3) | 0.87 (0.65-1.15) | 0.82 (0.56-1.19) | 0.88 (0.67-1.14) | 0.86 (0.58-1.28) |
| Low Birth weight | 2188 (71.7) | 83 (33.7) | 0.78 (0.59-1.03) | 0.67 (0.50-0.90) | 0.57 (0.38-0.87) | 0.67 (0.51-0.89) | 0.67 (0.44-1.01) |

BWDT, birth weight discordance in twins; CIs, confidence intervals; GA, gestational age; IPTW, inverse probability treatment weighting; OR, odds ratios; OW, overlap weighting; PSM, propensity score-matched.

**Table S9. The results of OR (95% CIs) for comparisons between prepregnancy normal weight and overweight in each model.**

|  | **Normal weight (n=3053)** | **Overweight**  **(n=877)** | **OR (95%CIs)** | | | | |
| --- | --- | --- | --- | --- | --- | --- | --- |
|  |  |  | **Crude** | **Adjusted** | **PSM** | **IPTW** | **OW** |
| **Total** (n/%) |  |  |  |  |  |  |  |
| GA <37 weeks | 1909 (62.5) | 559 (63.7) | 1.05 (0.90-1.23) | 0.96 (0.81-1.14) | 1.00 (0.81-1.23) | 0.97 (0.84-1.12) | 0.96 (0.76-1.22) |
| GA <34 weeks | 483 (15.8) | 175 (20.0) | 1.33 (1.09-1.60) | 1.28 (1.03-1.58) | 1.15 (0.88-1.50) | 1.27 (1.06-1.52) | 1.29 (0.95-1.75) |
| BWDT ≥20% | 520 (17.0) | 148 (16.9) | 0.99 (0.81-1.21) | 0.96 (0.78-1.17) | 1.02 (0.79-1.31) | 0.95 (0.79-1.13) | 0.95 (0.72-1.27) |
| **Larger twin** |  |  |  |  |  |  |  |
| 1 min Apgar ≤ 7 | 155 (5.1) | 67 (7.6) | 1.55 (1.14-2.07) | 1.60 (1.17-2.18) | 1.36 (0.92-2.03) | 1.64 (1.25-2.14) | 1.64 (1.03-2.65) |
| 5 min Apgar ≤ 7 | 24 (0.8) | 12 (1.4) | 1.75 (0.84-3.45) | 1.70 (0.8-3.44) | 1.60 (0.61-4.49) | 1.79 (0.95-3.27) | 1.69 (0.57-5.57) |
| NICU admission | 1426 (46.7) | 412 (47.0) | 1.01 (0.87-1.17) | 0.96 (0.81-1.12) | 0.93 (0.76-1.14) | 0.96 (0.84-1.10) | 0.96 (0.76-1.20) |
| Low Birth weight | 1365 (44.7) | 361 (41.2) | 0.87 (0.74-1.01) | 0.78 (0.66-0.92) | 0.72 (0.59-0.87) | 0.79 (0.68-0.90) | 0.78 (0.62-0.99) |
| **Smaller twin** |  |  |  |  |  |  |  |
| 1 min Apgar ≤ 7 | 159 (5.2) | 64 (7.3) | 1.43 (1.05-1.93) | 1.43 (1.04-1.94) | 1.26 (0.85-1.89) | 1.44 (1.09-1.88) | 1.45 (0.91-2.32) |
| 5 min Apgar ≤ 7 | 24 (0.8) | 13 (1.5) | 1.90 (0.94-3.69) | 1.63 (0.78-3.25) | 1.69 (0.66-4.60) | 1.68 (0.91-3.02) | 1.63 (0.58-5.01) |
| NICU admission | 1536 (50.3) | 452 (51.5) | 1.05 (0.90-1.22) | 0.97 (0.83-1.14) | 0.91 (0.75-1.12) | 0.98 (0.85-1.12) | 0.97 (0.77-1.22) |
| Low Birth weight | 2188 (71.7) | 607 (69.2) | 0.89 (0.76-1.05) | 0.80 (0.67-0.95) | 0.78 (0.63-0.97) | 0.80 (0.69-0.93) | 0.80 (0.62-1.02) |

BWDT, birth weight discordance in twins; CIs, confidence intervals; GA, gestational age; IPTW, inverse probability treatment weighting; OR, odds ratios; OW, overlap weighting; PSM, propensity score-matched.
