# Supplementary figures and images for: Maternal prepregnancy overweight/obesity increase the risk of low Apgar scores in twins: a population-based cohort study in China
Source: Front Pediatr. 2025 Jan 17;12:1412975. doi: 10.3389/fped.2024.1412975 (PMC11782157; doi:10.3389/fped.2024.1412975)

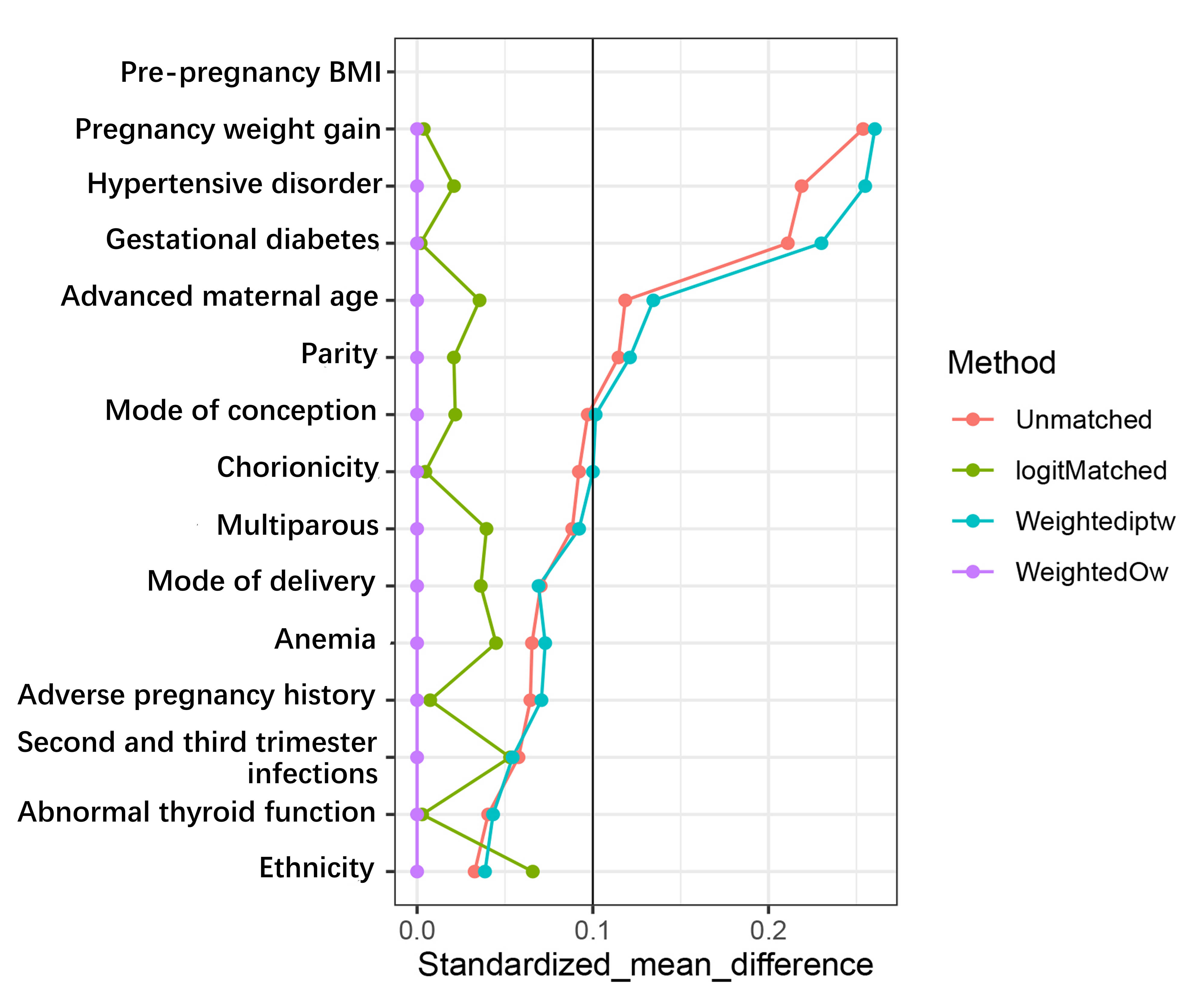

Supplement: Supplementary Figure S1 — Love plot of baseline variable balance between normal weight and overweight/obesity groups. This Love plot visualizes the standardized mean difference (SMD) values for baseline variables between normal weight and overweight/obesity groups in unmatched and adjusted models, including propensity score matching (PSM), inverse probability treatment weighting (IPTW), and overlap weighting (OW). Variables assessed include prepregnancy BMI, pregnancy weight gain, hypertensive disorder, gestational diabetes, advanced maternal age, parity, mode of conception, chorionicity, multiparous status, mode of delivery, anemia, adverse pregnancy history, infections in the second and third trimesters, abnormal thyroid function, and ethnicity. An SMD <10% indicates a relatively good balance between groups. [file Image1.jpeg]

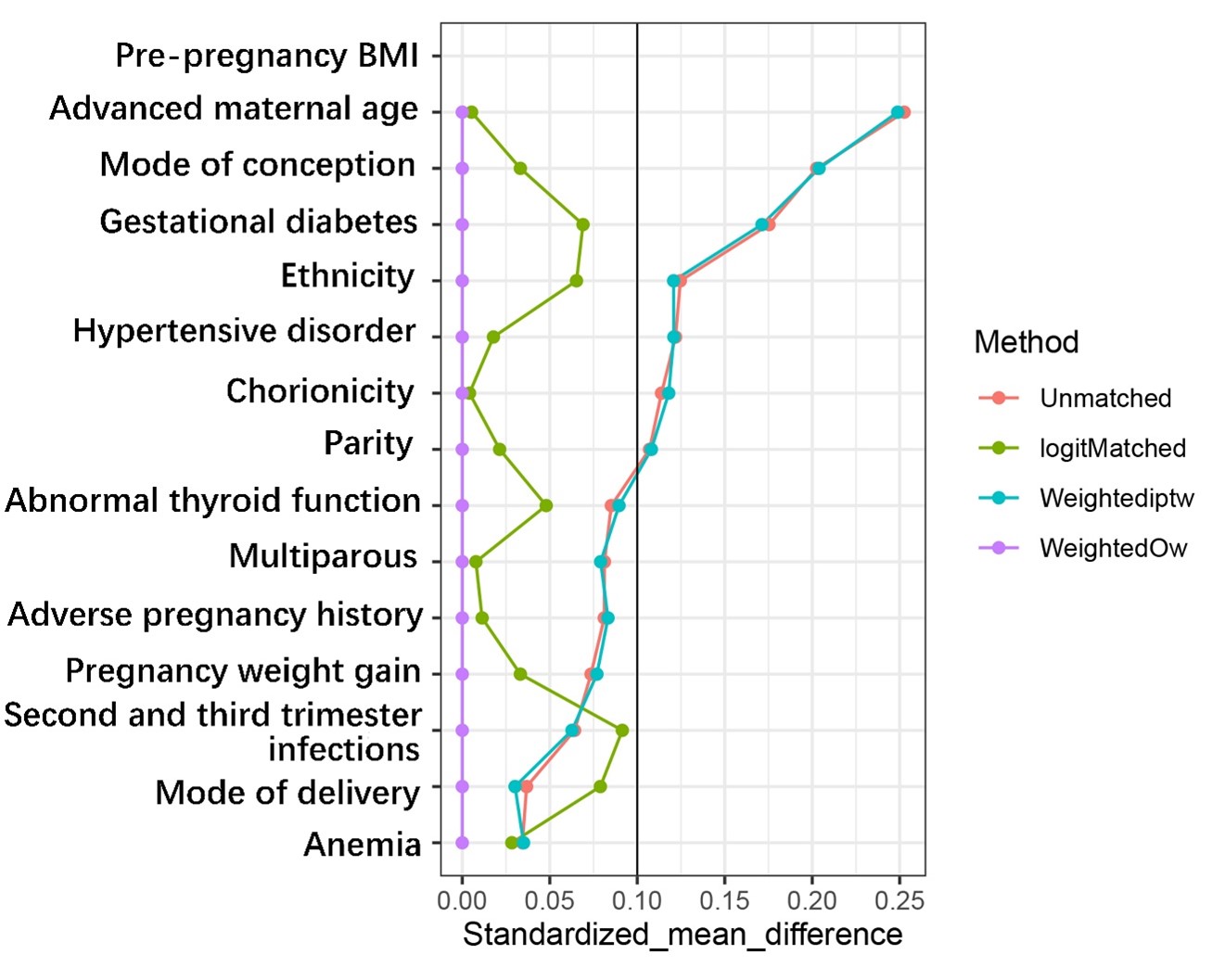

Supplement: Supplementary Figure S2 — Love plot of baseline variable balance between underweight and normal weight groups. This Love plot depicts the standardized mean difference (SMD) values for baseline variables between underweight and normal weight groups across different models, including the unmatched model and adjusted models: propensity score matching (PSM), inverse probability treatment weighting (IPTW), and overlap weighting (OW). The key variables assessed are prepregnancy BMI, advanced maternal age, mode of conception, gestational diabetes, ethnicity, hypertensive disorders, chorionicity, parity, abnormal thyroid function, multiparity, adverse pregnancy history, pregnancy weight gain, second- and third-trimester infections, mode of delivery, and anemia. An SMD of less than 10% signifies a relatively good balance between the groups. This analysis demonstrates the effectiveness of these models in achieving covariate balance for more reliable comparisons. [file Image2.jpeg]
